# LINE-1 hypomethylation in normal colon mucosa is associated with poor survival in Chinese patients with sporadic colon cancer

**DOI:** 10.18632/oncotarget.4450

**Published:** 2015-06-29

**Authors:** Changhua Zhuo, Qingguo Li, Yuchen Wu, Yiwei Li, Jia Nie, Dawei Li, Junjie Peng, Peng Lian, Bin Li, Guoxiang Cai, Xinxiang Li, Sanjun Cai

**Affiliations:** ^1^ Department of Colorectal Surgery, Fudan University Shanghai Cancer Center, Department of Oncology, Shanghai Medical College, Fudan University, Shanghai 200032, People's Republic of China; ^2^ Department of Surgical Oncology, Fujian Provincial Cancer Hospital, Teaching Hospital of Fujian Medical University, Fuzhou 350014, People's Republic of China; ^3^ Key Laboratory of Molecular Virology & Immunology, Unit of Molecular Immunology, Institut Pasteur of Shanghai, Shanghai Institutes for Biological Sciences, Chinese Academy of Sciences, Shanghai 200031, People's Republic of China

**Keywords:** colon cancer, LINE-1, microsatellite instability, epigenetic modification, survival analysis

## Abstract

Genetic and epigenetic pathways are not independent in colorectal cancer (CRC) carcinogenesis. We aimed to determine the influence of various molecular features on Chinese patients' colon cancer-specific survival (CCSS). Various genetic and epigenetic modifications were detected in paired tumor and normal mucosa tissue samples. The prognostic variables regarding patient CCSS were determined. Overall, 127 patients, including 83 males and 44 females, completed a median follow-up of 65 (3–85) months. A mean LINE-1 methylation rate of 64.62% (range, 9.45–86.93) was observed. Hypermethylation at the hMLH1 gene promoter was detected in 26 (20.47%) patients. *KRAS* was mutated in 52 (40.94%) patients. Sixteen (12.60%) patients were confirmed as microsatellite instability (MSI)-High, and 76 (59.84%) were found to have loss of heterozygosity at 18q. The LINE-1 methylation level, MSI status, perineural invasion and distant metastases were confirmed as independent prognostic factors for patient CCSS. A stratified survival analysis further revealed that certain subgroups of patients with LINE-1 hypomethylation had significantly worse survival (all *p* < 0.05). Our data revealed that both genetic and epigenetic abnormalities can concurrently exist during colonic tumorigenesis. As a global epigenetic change, LINE-1 hypomethylation in normal colon mucosa might be associated with a worse outcome in certain Chinese patients with colon cancer.

## INTRODUCTION

Colorectal cancer (CRC) is one of the most common malignancies in the United States and worldwide [1]. Three characteristics have been implicated in CRC tumorigenesis: chromosomal instability (CIN), microsatellite instability (MSI), and the CpG island methylator phenotype (CIMP) [2]. CRC can evolve through the classical adenoma-carcinoma sequence or the alternative serrated pathway [3]. The genetic basis of sporadic CRC has been an intensely studied topic in the field of cancer biology over the past three decades [4]. The adenoma–carcinoma sequence is the main pathway for CRC development and is characterized by carcinoma with microsatellite stability (MSS) and CIN. The consequence of CIN may be a higher frequency of loss of heterozygosity (LOH) [5]. In this pathway, an ordered series of events occurs, starting with the transformation of normal epithelium into aberrant crypt foci and followed by the development of transitional adenoma and finally adenocarcinoma [6]. This progression involves the initial inactivating mutation in the APC gene, sequential activating mutations in the *KRAS* and *PIK3CA* genes and inactivating mutations in the *DCC, SMAD2/SMAD4* and *TP53* genes at different stages of tumorigenesis [5–9].

CRC encompasses a heterogeneous group of diseases that may arise from epigenetic alterations as well [10]. MSI occurs in approximately 15% of sporadic CRCs, usually through the serrated pathway [11–13]. The CIMP develops early in this sequence, and CIMP tumors seem to be strongly associated with the BRAF V600E mutation [13–17]. Unlike Lynch syndrome, sporadic carcinoma with MSI arises as a result of the inactivation of DNA mismatch repair (MMR) genes, such as *MLH1*, through promoter hypermethylation [5].

DNA methylation is the major epigenetic mechanism responsible for X-chromosome inactivation, imprinting, and the repression of endogenous retroviruses [18, 19]. It is well established that genome-wide hypomethylation occurs in tumors, and the overexpression of oncogenes has been suggested to be the result of this hypomethylation [20–23]. The human genome contains transcriptionally inactive non-coding DNA elements, including long interspersed nuclear element-1 (LINE-1) repetitive sequences [24–26].

LINE-1 contains numerous CpG dinucleotides, and studies have shown that the level of LINE-1 methylation is a good indicator of cellular 5-methylcytosine levels (i.e., global DNA methylation levels) [27–29]. Hypomethylation of global LINE-1 DNA elements is associated with CIN [30, 31]. LINE-1 hypomethylation in the normal mucosa of CRC patients has been observed and reported to be significantly associated with poor prognosis [23, 32]. Thus, the hypomethylation of LINE-1 in adjacent normal mucosa may play an important role in forming a “field defect” and in influencing the progression of colorectal carcinogenesis [27, 33–36].

This study aimed to first investigate the clinicopathological characteristics and molecular alterations, including genetic and epigenetic changes, in Chinese patients with sporadic colon cancer at a single center. Second, we sought to determine the prognostic variables for colon cancer-specific survival (CCSS). Finally, we aimed to determine whether LINE-1 hypomethylation in the adjacent normal mucosa constitutes a methylation “field defect”, which may influence patient survival.

## RESULTS

### Patient characteristics

A total of 127 patients, 83 males and 44 females, were included in the present study. These patients completed a median follow-up of 65 (3–85) months. The patient characteristics and clinicopathological features are presented in Table 1.

**Table 1:** Patients' characteristics

Clinicopathological variables		N	%
Sex	Male	83	65.4
	Female	44	34.6
Age (years)	≤60	62	48.8
	>60	65	51.2
Maximum Size (cm)	≤5	80	63.0
	>5	47	37.0
Gross Shape	Ulcerative type	83	65.4
	Protruded type	38	29.9
	Infiltrative type	6	4.7
Location^a^	Left-sided	56	44.1
	Right-sided	71	55.9
Differentiation	G1-G2	78	61.4
	G3-G4	49	38.6
Mucinous or signet-ring carcinoma	No	102	80.3
	Yes	25	19.7
Serum CEA level	Normal	78	61.4
	Elevated	49	38.6
Serum CA199 level	Normal	86	67.7
	Elevated	41	32.3
Tumor stage (T)	T1	0	0.0
	T2	17	13.4
	T3	36	28.3
	T4a	65	51.2
	T4b	9	7.1
Nodal status (N)	N0	68	53.5
	N1a	5	3.9
	N1b	20	15.7
	N1c	10	7.9
	N2a	8	6.3
	N2b	16	12.6
Distant metastases (M)	M0	94	74.0
	M1	33	26.0
AJCC stage	I	16	12.6
	II	45	35.4
	III	33	26.0
	IV	33	26.0
Lymphovascular invasion	No	85	66.9
	Yes	42	33.1
Perineural invasion	No	109	85.8
	Yes	18	85.8
Extranodal tumor deposits	No	108	85.0
	Yes	19	15.0

a The left side of the colon consists of the splenic flexure, descending, and sigmoid colon. The right side of the colon consists of the cecum, ascending colon, hepatic flexure, and transverse colon.

Abbreviations: CEA, carcinoembryonic antigen; CA199, Carbohydrate antigen 199; AJCC, American Joint Committee on Cancer.

### LINE-1 methylation levels in mucosa adjacent to the tumor nest

**Figure 1:**
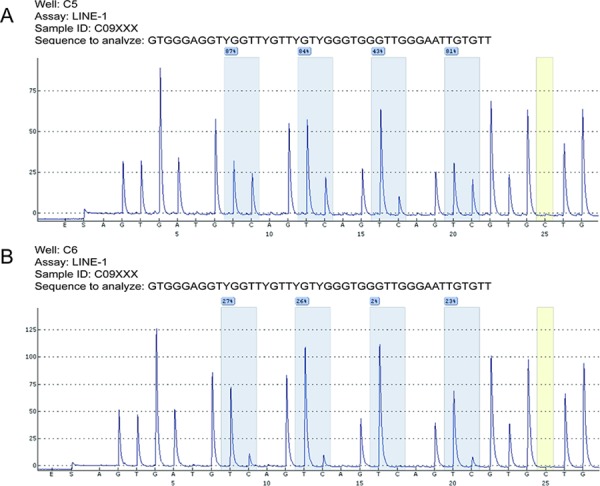
Representative LMR results after pyrosequencing Bisulfite-treated DNA samples from adjacent normal mucosa were subjected to PCR amplification and were quantitatively analyzed by pyrosequencing. The C base marked in yellow served as a quality control of the bisulfite conversion efficiency. Four analyzed CpG sites are highlighted in blue, and the percent methylation rate is provided for each site. The mean percentage was computed as the LINE-1 methylation rate (LMR) for each case. Two cases with relatively higher (73.8%, **A.**) or lower (19.4%, **B.**) LMR were shown, respectively.

**Figure 2:**
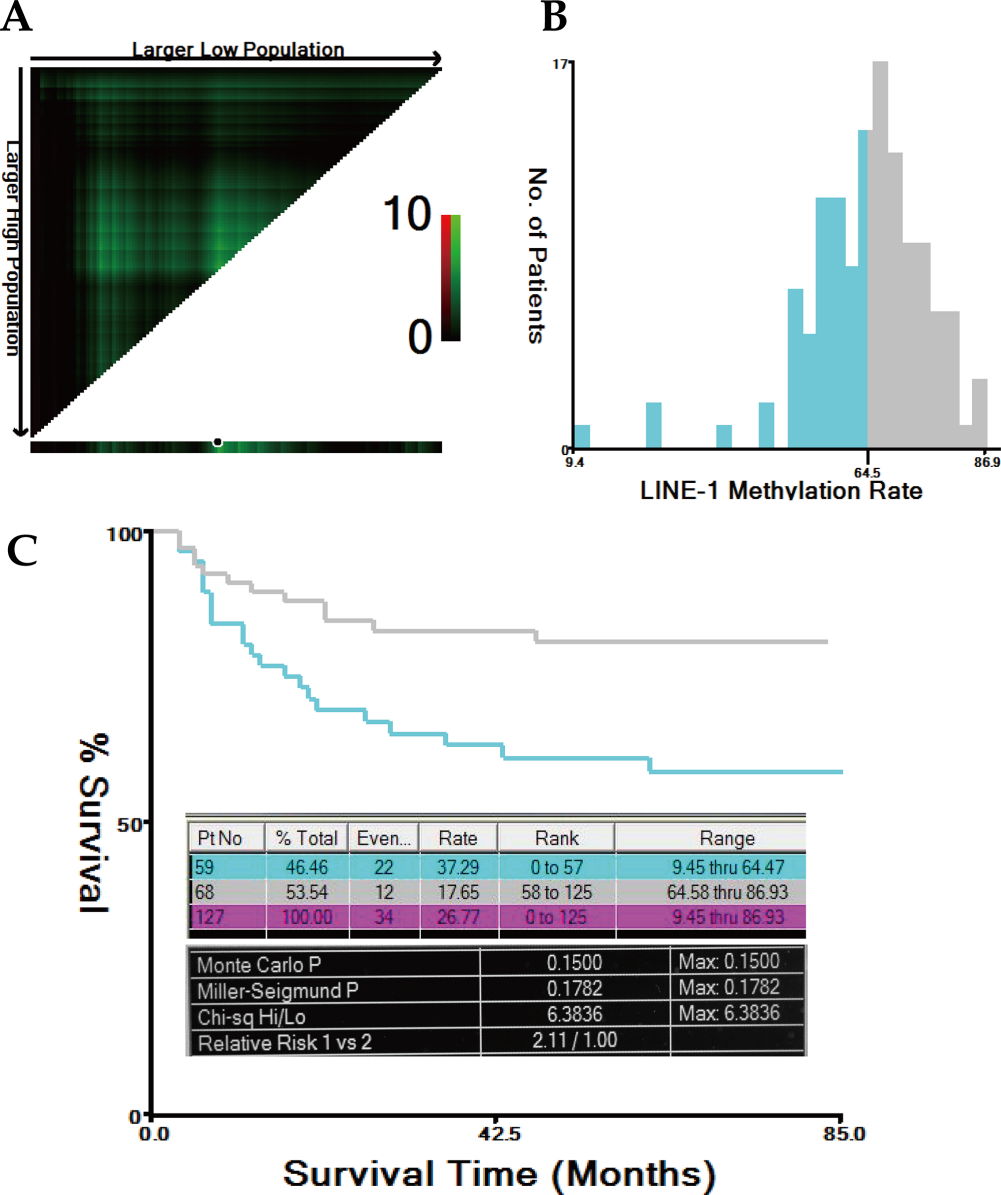
Cutoff value for LMR calculated using the X-tile program The X-tile program was utilized to calculate the optimal cutoff value for the LINE-1 methylation rate (LMR). Based on the patient survival data, the entire population was divided into the training and validation sets. The training set is shown in the upper-left quartile, with plots of the matched validation set in the small long strip (on the bottom X-axis). The black dot in the validation set represents the exact cutoff value for the LMR **(A).** The entire cohort was divided into low (blue) or high (grey) LMR groups based on the cutoff value (64.47%), as shown in the histogram **(B).** Kaplan-Meier plots were generated based on this cutoff value. The detailed outputs of the X-tile analysis are presented (maximum high/low x^2^ = 6.38, Monte Carlo *P* = 0.15) **(C).**

A mean LINE-1 methylation rate (LMR) of 64.62% (range, 9.45–86.93%; standard deviation, 11.72%) was determined by pyrosequencing. Representative results are shown in Figure 1. The LMRs in the 127 normal colonic mucosa samples were normally distributed (Kolmogorov-Smirnov *Z* = 0.881; *p* = 0.4200) (Supplementary Table S1, available online). Using the X-tile program, the patients were subgrouped into two populations based on a high or low LMR with a cutoff value of 64.47% (maximum x^2^ = 6.38; *p* = 0.15; Figure 2).

### Hypermethylation at the *hMLH1* and *hMSH2* promoters

The median percentage of methylated reference (PMR) of the analyzed CpG islands at the *hMLH1* and *hMSH2* promoters were determined by methylation-specific quantitative polymerase chain reaction (MS-qPCR) to be 0.13% (range, 0.01–93.67%) and 2.39% (range, 0.17–7.57%), respectively. Twenty-six (20.47%) and 19 (14.96%) patients were determined to have hypermethylation at the *hMLH1* and *hMSH2* promoters, respectively.

### Gene mutational analysis

The most common mutations occurred in the *KRAS* gene, which was mutated in 52 of the 127 cases (40.94%). The other gene mutations included the following: 5 (3.94%) in *BRAF*, 3 (2.36%) in *NRAS*, and 7 (5.51%) in *PIK3CA*. The mutation analysis results are shown in Table 2. The chi-square test revealed a significantly higher mutation rate in the *KRAS* gene in right-sided tumors compared to left-sided tumors (50.7% vs. 28.6%, x^2^ = 6.342, *p* = 0.012; Supplementary Table S2, available online).

**Table 2:** Gene mutations result analyzed by Sanger sequencing

Gene	Analyzed mutation points	N of mutation	Codon	Subtotal in codon	Total	Overall mutation rate (%)
*BRAF*	c.1798G>A/T	0	V600	5	5	3.94
	c.1799T>A	4				
	c.1799T>G	1				
	c.1799T>C	0				
*KRAS*	c.34G>A/C/T	10	G12	38	52	40.94
	c.35G>A/C/T	28				
	c. 37G>A/C/T	1	G13	10		
	c. 38G>A/C/T	9				
	c. 181C>A/G/T	0	Q61	2		
	c. 182A>C/G/T	0				
	c. 183A>C/T	2				
	c.436G>A/C	1	A146	2		
	c.437C>T	1				
*NRAS*	c. 34G>A/C/T	1	G12	2	3	2.36
	c. 35G>A/C/T	1				
	c.37G/38G>A/C/T	0	G13	0		
	c.181C>A/G/T	0	Q61	1		
	c. 182A>C/G/T	1				
	c.183A>C/T	0				
*PIK3CA*	c.1624G>A/C	2	E542	2	7	5.51
	c.1633G>A/C	0	E545	0		
	c.1636C>A/G	1	E546	2		
	c.1637A>C/G/T	1				
	c.3139C>T	0	H1047	3		
	c.3140A>G/T	3				
	c.3145G>A/C	0	G1049	0		

Abbreviations: BRAF, v-raf murine sarcoma viral oncogene homolog B; KRAS, Kirsten rat sarcoma viral oncogene homolog; NRAS, neuroblastoma RAS viral oncogene homolog; PIK3CA, phosphatidylinositol-4, 5-bisphosphate 3-kinase, catalytic subunit a.

### MSI and 18q LOH status analysis

**Figure 3:**
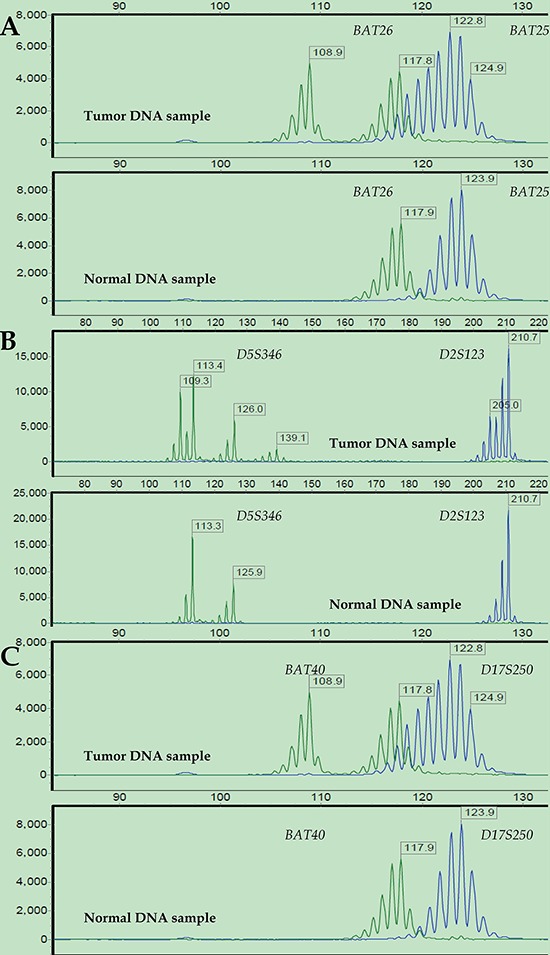
Representative MSI status results after STR analysis Electropherograms of labeled PCR products targeting six microsatellite loci in paired tumor (upper) and normal (bottom) DNA samples from a representative patient: *BAT26* and *BAT25* **(A)**, *D5S346* and *D2S123* **(B)**, and *BAT40* and *D17S250* **(C).** The PCR product size is represented on the X-axis, and fluorescence units are represented on the Y-axis. For all the microsatellite loci, the tumor DNA sample showed altered allelic profiles compared to the matched normal DNA sample. Thus, this case was defined as MSI-H.

**Figure 4:**
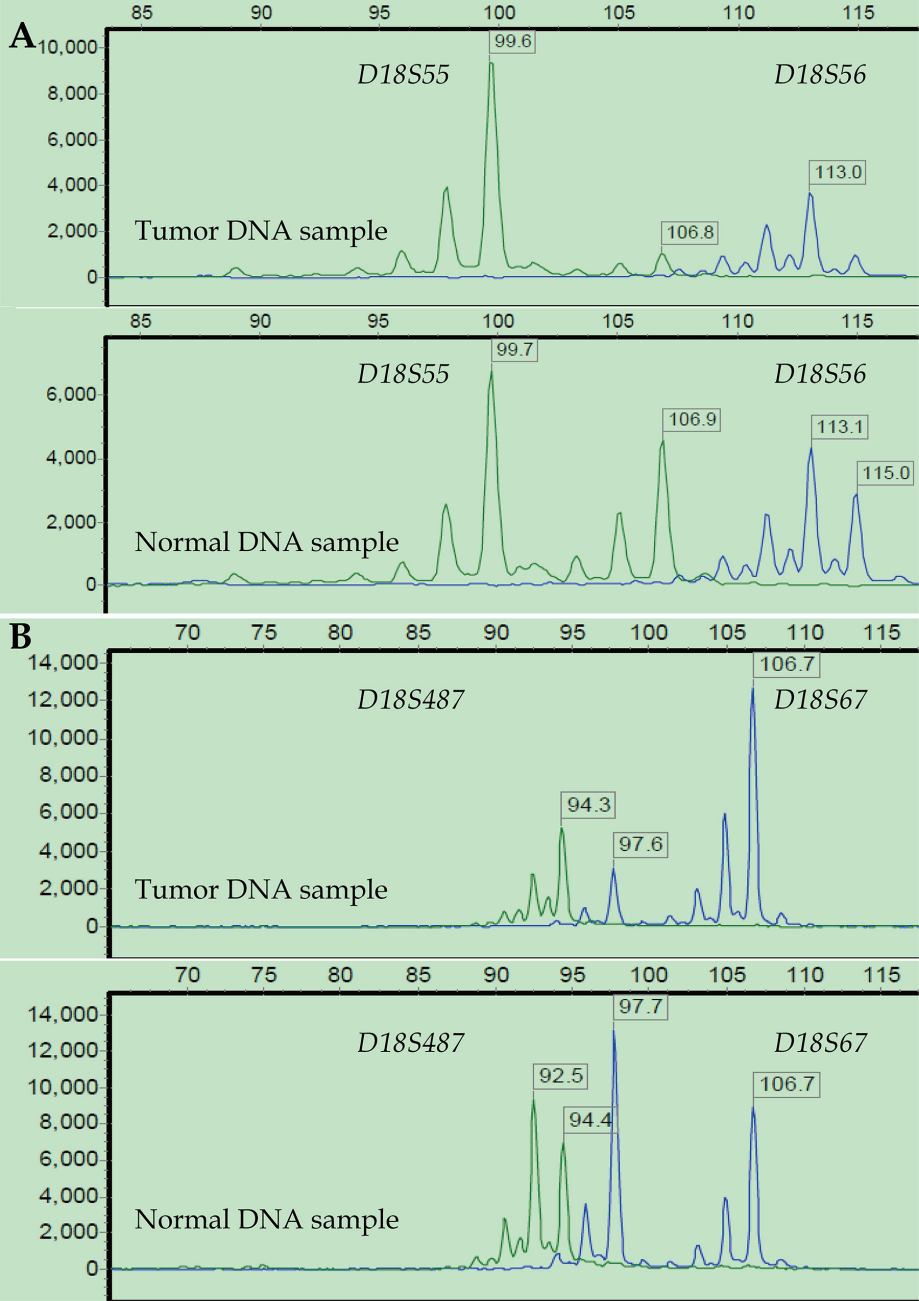
Representative 18q LOH status results after STR analysis LOH status was investigated at four loci on 18q from a representative patient: *D18S55* and *D18S56* **(A)**, *D18S67* and *D18S487* **(B).** Three loci (*D18S55, D18S56*, and *D18S487*) showed a greater than 40% reduction in fluorescence units in at least 1 of 2 allele peaks in tumor DNA relative to normal DNA. Thus, this tumor was defined as 18q LOH-positive, and MSI-High as well.

The short tandem repeat (STR) analysis confirmed 16 (12.60%), 40 (31.50%), and 71 (55.90%) cases as MSI-High (MSI-H), MSI-Low (MSI-L) and MSS, respectively. The chi-square test revealed a significantly higher hypermethylation rate of the *hMLH1* promoter in the subpopulation of MSI-H tumors compared to that of MSI-L/MSS tumors (36.4% vs. 17.1%, x^2^ = 4.127, *p* = 0.042; Supplementary Table S3, available online). In the 18q LOH status analysis, 76 (59.84%) cases were LOH-positive at chromosome 18q. The representative results of the STR analysis of MSI and 18q LOH are shown in Figures 3 and 4.

### Kaplan-Meier survival and multivariate Cox regression analyses

The Kaplan-Meier survival analysis revealed that tumor stage (T), nodal status (N), distant metastases (M), AJCC stage, sex, LMR, MSI status, 18q LOH, serum CEA and CA199 levels, lymphovascular invasion, extranodal tumor deposits and perineural invasion significantly influenced patients' CCSS (all *p* < 0.05; Table 3). The multivariate Cox regression analysis confirmed that the LMR (high vs. low, hazard ratio (HR) = 0.337, 95% confidence interval (CI): 0.162–0.702, *p* = 0.004), MSI status (MSI-H vs. MSI-L/MSS, HR = 0.088, 95% CI: 0.011–0.679, *p* = 0.020), perineural invasion (yes vs. no, HR = 2.578, 95% CI: 1.148–5.791, *p* = 0.022), and distant metastases (M1 vs. M0, HR = 28.641, 95% CI: 11.414–71.870, *p* = 0.000) were independent prognostic factors of CCSS (Table 4).

**Table 3:** Kaplan-Meier analysis on patient's CCSS

Prognostic variable	Grouping factor	Mean (months)	SE (months)	95% CI (lower-upper)	X^2^	*p*
Sex	Male	69.670	3.319	63.164–76.175	6.278	0.012
	Female	54.911	5.216	44.689–65.134		
T stage	T2–T3	74.363	3.495	67.513–81.214	8.215	0.004
	T4a–T4b	56.711	4.061	48.751–64.670		
N stage	N0	77.898	2.392	73.209–82.588	22.908	0.000
	N1–N2	48.913	4.950	39.211–58.614		
M stage	M0	79.188	1.905	75.455–82.922	107.858	0.000
	M1	19.610	4.043	11.685–27.535		
AJCC TNM Stage	I–II	81.395	1.758	77.949–84.841	30.134	0.000
	III–IV	48.409	4.624	39.347–57.472		
LMR Level	Low	57.414	4.679	48.243–66.585	5.582	0.018
	High	69.692	3.376	63.076–76.309		
MSI Status	MSI–L/MSS	60.766	3.317	54.264–67.267	6.380	0.012
	MSI–H	81.895	3.022	75.971–87.819		
18q LOH	No	72.341	3.900	64.696–79.986	4.258	0.039
	Yes	59.258	3.968	51.482–67.035		
Serum CEA level	Normal	70.678	3.304	64.202–77.154	7.606	0.006
	Elevated	55.259	5.117	45.231–65.288		
Serum CA199 level	Normal	75.834	2.587	70.762–80.905	37.850	0.000
	Elevated	38.593	5.144	28.511–48.674		
LVI	No	71.556	3.060	65.558–77.554	10.980	0.001
	Yes	50.982	5.675	39.859–62.105		
PNI	No	68.083	2.934	62.332–73.834	7.805	0.005
	Yes	45.389	8.922	27.903–62.875		
ENTD	No	69.139	2.882	63.490–74.788	16.714	0.000
	Yes	33.748	6.840	20.341–47.154		

*Log Rank (Mantel-Cox) test was used to test the significance of the different survival between the groups according to different variables. A two-tailed *p* value ≤0.05 was considered statistically significant.

Abbreviations: CCSS, colon cancer specific survival; SE, standard error; CI, confidence interval; AJCC, American Joint Committee on Cancer (AJCC); LMR, LINE-1 methylation rate; 18q LOH, loss of heterozygosity at chromosome 18q; MSI, micro-satellite instable; MSS, micro-satellite stable; CEA, carcinoembryonic antigen; CA199, Carbohydrate antigen 199; LVI, lymphovascular invasion; PNI, perineural invasion; ENTD, extranodal tumor deposits.

**Table 4:** Multivariate Cox analysis on prognostic factors for patient's CCSS

Variables	SE	Wald	*p*	HR	95.0% CI	
					Lower	Upper
Distant metastases	0.469	51.082	0.000	28.641	11.414	71.870
LMR level	0.374	8.457	0.004	0.337	0.162	0.702
MSI-H	1.041	5.435	0.020	0.088	0.011	0.679
Perineural invasion	0.413	5.262	0.022	2.578	1.148	5.791

Abbreviations: CCSS, colon cancer specific survival; SE, standard error; HR: hazard ratio; CI, confidence interval; LMR, LINE-1 methylation rate; MSI, micro-satellite instable.

### Stratified analysis of the influence of LMR on patient survival rate

**Figure 5:**
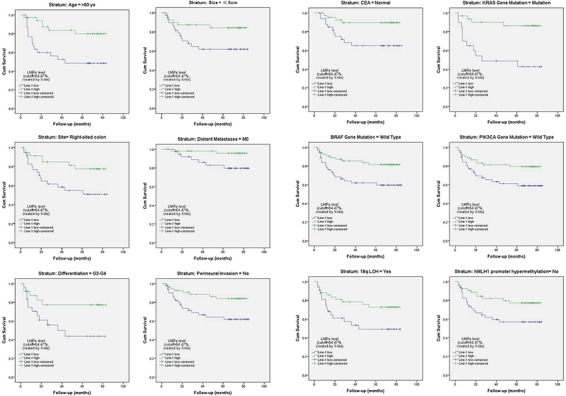
Stratified analysis of the influence of the LMR level on colon cancer-specific survival Kaplan-Meier survival studies were used for a stratified analysis. It revealed that patients with a lower LMR (LINE-1 hypomethylation) had a significantly worse survival rate among certain subgroups of patients with colon cancer (all *p* < 0.05; also see Supplementary Table S4, available online).

A stratified Kaplan-Meier survival analysis further revealed that patients with a lower LMR had a significantly worse survival in the subgroups of age >60 years, tumor size ≤5 cm, right-sided tumors, M0, differentiation grade of G3-G4, no perineural invasion, normal serum CEA levels, *KRAS* gene mutation, wild-type *BRAF* and *PIK3CA*, 18q LOH, and no *hMLH1* gene promoter hypermethylation (all *p* < 0.05; Figure 5; Supplementary Table S4, available online).

### Associations between LMR and other variables

The normality tests revealed that the LMRs in most of the subgroups were normally distributed according to various clinicopathological variables (most with *p* > 0.05; Supplementary Table S1, available online). Thus, the mean differences between the different subgroups were evaluated using Student's *t* test. However, these variables were not associated with the LMR (all *p* > 0.05; Supplementary S5, available online). The remaining two variables, *PIK3CA* gene mutation and lymphovascular invasion, were not normally distributed but were associated with the LMR (Mann-Whitney *U* test, all *p* < 0.05; Table 5).

**Table 5:** Mann-Whitney *U* test for association between LMRs and certain variables

Variables	Subgroup	*N*	Mean rank	Sum rank	U	*p*
Lymphovascular invasion	No	85	69.88	5939.50	1285.500	0.010
	Yes	42	52.11	2188.50		
*PIK3CA* gene mutation	Wild type	120	62.23	7467.00	207.000	0.024
	Mutation	7	94.43	661.00		

*A two-tailed *p* value ≤ 0.05 was considered statistically significant.

## DISCUSSION

LINE-1 methylation levels have been reported to be a surrogate marker for cellular 5-methylcytosine levels (i.e., global DNA methylation) [23, 28, 29, 37–39]. Herein, we investigated the relationship between the survival of patients with colon cancer and global DNA methylation levels in normal colonic mucosa as well as various other molecular alterations. Our findings revealed that LINE-1 hypomethylation in normal-appearing mucosa was significantly associated with worse survival in certain subgroups of Chinese patients with colon cancer. This association has also been reported in other ethnic groups [27, 33, 34, 40].

CRC consists of a heterogeneous group of diseases with complex genetic and epigenetic modifications [41]. Genetic alterations usually involve mutations in oncogenes and/or tumor suppressor genes that result in either a gain or loss of function and abnormal expression. The consequence of such alterations is the aberrant activation or repression of downstream genes governing cell proliferation and growth [42]. Epigenetic alterations that contribute to CRC tumorigenesis are more complex and usually involve chromatin structural modifications such as histone modifications, aberrant DNA methylation, and nucleosome positioning [7, 43]. In the present study, we conducted an overall investigation of the potential factors that influence the prognosis of patients with colon cancer with a particular focus on genetic (somatic mutations and CIN/18q LOH) and epigenetic (LINE-1 hypomethylation, *hMLH1* and *hMSH2* promoter hypermethylation, and MSI status) changes and correlated these changes with certain established clinicopathological features.

The majority of CRCs that occur via the adenoma–carcinoma sequence have distinct features with genetic mutations in various oncogenes and tumor suppressor genes [43]. Somatic mutations in *KRAS* are common in CRC [44]. In the present study, the *KRAS* gene mutation rate (40.94%) was comparable to that reported by others [45, 46]. Among the 52 cases with mutant *KRAS*, the majority had mutations at codons G12 and G13 (38 and 10 cases, respectively). *NRAS* mutations are rare in CRC [47]. We only detected 3/127 (2.36%) cases of mutant *NRAS* (Table 2). Furthermore, we found an increased incidence of *KRAS* mutation in tumors located in the proximal colon (Supplementary Table S2). This result was also in accordance with those of other studies [45, 48, 49]. Interestingly, we found a total of 60 (47%) tumors with mutated *KRAS*, *NRAS* or *BRAF* genes, and the significant pattern of mutual exclusivity among these genes has been reported previously [50, 51]. However, the exact mechanism for this mutual exclusivity is not yet clear.

In addition to contributing to genetic mutations, CIN contributes to the pathogenesis of conventional CRC that develops via the adenoma-carcinoma sequence [52]. LOH is considered to be a hallmark of CIN-positive tumors [5]. Fearon et al. [53] originally determined that the evolution of CRC was frequently associated with mutated genes on chromosome 18q. In the present study, 76 (59.84%) tumors were LOH-positive at chromosome 18q. This finding agrees with the results of a study by Thiagalingam et al. [54]. The authors conducted a cytogenetic analysis of LOH at chromosomes 1, 5, 8, 17, and 18 in patients with CRC and concluded that LOH was common at chromosome 18, which appeared to be caused by mitotic recombination or gene conversion.

The serrated pathway that occurs in colorectal carcinogenesis is predominantly influenced by epigenetic modifications and characterized by *BRAF* mutations [5, 55]. However, activating mutations in *BRAF* are less common in CRC [56]. We detected *BRAF* mutations in only 5 (3.94%) tumors, and all of these mutations occurred in codon V600 (c.1799T > A/G) (Tables 2 and Supplementary Table S6).

Epigenetic modifications can also cause MMR gene silencing and thus predispose a cell to *hMLH1* inactivation via promoter hypermethylation [2, 43]. These observations may explain why sporadic CRC that develops via the serrated pathway has a distinct potential endpoint as a MSI carcinoma [5]. In our cohort, we detected 40 (31.50%) and 16 (12.60%) cases that were MSI-H and MSI-L, respectively. Hypermethylation of MMR genes and LINE-1 DNA elements in the normal mucosa of patients with CRC has been reported to be consistently detected [23, 31, 32, 57, 58]. Our data also confirmed a higher hypermethylation level at the *hMLH1* gene promoter in MSI-H tumors than in MSI-L or MSS tumors (Supplementary Table S2).

The CIMP is another distinct form of epigenomic instability in CRC that develops via the serrated pathway [59–63]; the CIMP causes most cases of sporadic CRC with MSI-H through epigenetic silencing of *hMLH1* [64, 65]. A CIMP-high status in CRC patients is regarded as a surrogate for the widespread hypermethylation of CpG islands [66, 67]. Previous CRC studies have identified associations between a CIMP-high status and a female preponderance, proximal colon location, MSI-H, increased age and *KRAS* mutation rate, or decreased *TP53* mutation rate [48, 49, 68–71]. However, we could not confirm these relationships with our own CIMP results (data not shown). A small sample size and a non-predominant mechanism of colorectal tumorigenesis via the serrated pathway potentially account for this inconsistency.

Genome-wide hypomethylation is a frequent somatic epigenetic alteration in cancer cells [72] and possibly contributes to a “field defect” in precancerous lesions [73]. Epigenetic and genetic changes apparently are not two separate mechanisms that participate in gastrointestinal carcinogenesis [43]. Our survival study showed that besides certain confirmed clinicopathological abnormalities, both genetic (18q LOH) and epigenetic (MSI and LMR) alterations contributed separately to the survival of patients with colon cancer (Table 3). Data from the multivariate Cox analysis reinforced the concurrent influence of genetic and epigenetic changes on patient survival (Table 4). Epigenetic alterations can cause genetic mutations, and *vice versa;* genetic mutations in epigenetic regulators can also lead to an altered epigenome [71]. Our data again confirmed this association between LINE-1 hypomethylation in normal mucosa and specific poor pathological features and genetic alterations (Table 5).

Suzuki et al. [74] found that hypomethylation was more strongly associated than hypermethylation with genetic damage and a worse prognosis. Similarly, Alonso et al. [75] reported an absence of an association between MGMT methylation and G > A transition mutations in *KRAS* and *TP53* in CRC without MSI. In the present study, we also did not identify a significant correlation between *hMLH1*/*hMSH2* hypermethylation and various gene mutations, regardless of MSI status.

One limitation of our study is that this relatively small, single center cohort included only Chinese participants. Thus, it remains to be determined whether our findings are applicable to general populations with CRC. Nonetheless, to the best of our knowledge, this was the first study aimed at investigating the prognostic significance of various genetic, epigenetic and clinicopathological variables on the survival of Chinese patients.

In conclusion, our data partially confirmed that genetic (classical adenoma-carcinoma sequence) and epigenetic (alterative serrated pathway) patterns can concurrently exist in the complex landscape of colonic tumorigenesis. Furthermore, LINE-1 hypomethylation in adjacent normal colon mucosa appeared to be associated with worse outcome in certain Chinese patients with colon cancer.

## MATERIALS AND METHODS

### Patients, tissue samples and clinicopathological variables

A total of 127 pairs of tissue samples were retrieved from patients with stage I-IV colon cancer. These consecutive patients were surgically treated by one medical team (Attending doctor, Prof. Sanjun Cai, M.D.) between January 2008 and December 2009. In this study, patients with resectable primary lesions, including those who had distant metastases that were either resectable or unresectable, were included. Patients who had received neoadjuvant chemotherapy and those with inflammatory bowel disease, familial adenomatous polyposis, Lynch syndrome, or serrated polyposis were excluded.

Fresh colon tumor tissues and paired normal colonic mucosa (at least 5 cm from the tumor margin) were obtained immediately after the specimens were retrieved in the operation room; these specimens were washed twice with chilled 1x phosphate-buffered saline, immediately frozen in liquid nitrogen, and stored at −80°C in our tissue bank for future use.

The patients' electronic medical records were reviewed, and various clinicopathological variables were investigated. Colon cancer differentiation grading and TNM classification were confirmed according to the criteria described in the AJCC Cancer Staging Manual (7th edition, 2010). The primary outcome of this study was CCSS, which was computed from the time when the patient underwent an operation until death from colon cancer. The last follow-up date was set as December 31, 2014. Written informed consent was obtained from all the patients, and the study protocol was approved by the Medical Ethics Committee of Fudan University Shanghai Cancer Center.

### Genomic DNA isolation and bisulfite conversion

Genomic DNA (gDNA) was isolated from tumor or normal colonic mucosa tissue samples using tissue DNA isolation kits (#D3051, ZYMO Research, USA) according to the manufacturer's instructions. gDNA was quantified using a spectrophotometer (NanoDrop 2000, Thermo Fisher Scientific Inc., USA). Bisulfite treatment of 0.5–1 μg of gDNA (tumor or normal mucosa) was performed using methylation kits (#D5006, ZYMO Research, USA) according to the manufacturer's instructions.

### Pyrosequencing for LINE-1 methylation levels

Bisulfite-treated DNA samples from normal colon mucosa were subjected to PCR amplification using an ABI GeneAmp^®^ PCR System 9700 (Applied Biosystems, USA); the 50-μL reactions contained 0.2 μL (5 U/μl) of KAPA Taq DNA Polymerase (Kapa Biosystems, USA), 50 pmol of each forward and reverse primer, and 2 μL of bisulfate-converted DNA. The PCR conditions were as follows: initial Taq activation at 95°C for 3 minutes; 40 cycles of denaturation at 94°C for 30 seconds, annealing at 50°C for 30 seconds, and elongation at 72°C for 1 minute; and a final extension at 72°C for 7 minutes. Global LINE-1 methylation levels were quantitatively analyzed using the PyroMark Q96 ID pyrosequencing system (Qiagen, German) as described previously [35, 36]. The mean percent methylation of the four analyzed CpG sites was calculated as the LMR. The primer sequences are provided in Supplementary Table S5 (available online).

### MS-qPCR for *hMLH1* and *hMSH2* promoter hypermethylation

Bisulfite-treated DNA samples from tumor tissues were analyzed for *hMLH1* and *hMSH2* hypermethylation. MS-qPCR (MethyLight) was performed using SYBR Green reagent (#K0221, Thermo Scientific, USA). In this system, a bisulfite-converted universal human DNA standard of 100% methylation (#D5015, ZYMO Research, USA) and *ALU-C4* were used as the reference template and internal control, respectively. Real-time PCR was performed in a final reaction volume of 10 μL using an ABI Prism 7900T Sequence Detection System (Applied Biosystems, USA). The reaction mixture contained 25 pmol of target gene primers (*hMLH1* or *hMSH2*) or control primers (*ALU-C4*) and 25–50 ng of bisulfite-treated sample DNA template or DNA standard. The cycling conditions were as follows: initial denaturation at 95°C for 10 minutes followed by 40 cycles of denaturation at 95°C for 15 seconds and annealing/extension at 60°C for 1 minute. The PMR was computed using a previously described formula [76]: 100% * 2 exp–[Delta Ct (target gene in sample − control gene in sample) − Delta Ct (100% methylated target in reference sample – control gene in reference sample)]. A PMR cutoff of 4%, which was previously validated [77–79], was utilized to determine whether a sample was hypermethylated at the *hMLH1* and *hMSH2* gene promoters. The primer sequences are provided in Supplementary Table S6 (available online).

### Sanger sequencing analysis of gene mutation status

In the present study, the gene mutation status of the most frequently reported CRC-related oncogenes, *BRAF*, *KRAS*, *NRAS*, and *PIK3CA*, was analyzed. Sanger sequencing was performed targeting *BRAF* codon 600; *KRAS* codons 12, 13, 61 and 146; *NRAS* codons 12, 13 and 61; and *PIK3CA* codons 542, 545, 546, 1047 and 1049. The possible point mutation sites and the primer sequences are listed for each gene in Supplementary Table S7 (available online).

Tumor tissue gDNA samples were analyzed to determine the mutation status of the aforementioned genes. Approximately 10 ng of gDNA was amplified in a 25-μL PCR reaction that contained 10 pmol of forward and reverse primers and 12.5 μL of KAPA2G Fast Multiplex Mix (#KM5802, Kapa Biosystems, USA). The thermocycling conditions were as follows: initial activation at 94°C for 5 minutes; 30 cycles of denaturation at 94°C for 30 seconds, annealing at 60°C for 30 seconds, and elongation at 72°C for 1 minute; and a final extension at 72°C for 5 minutes. The PCR products were extracted with a gel extraction kit (#AP-GX-250, Axygen Biosciences, USA) and purified using an ABI PRISM BigDye Reaction Kit (#403047, Applied Biosystems, USA) according to the manufacturer's instructions. After purification, the products were analyzed using an ABI 3730XL Genetic Analyzer (Applied Biosystems, USA). Specific point mutations were analyzed individually, and the overall mutation rate was calculated for each gene. A gene was defined as wild-type based on the absence of a point mutation at any of these sites.

### STR analysis for MSI and 18q LOH status

gDNA samples extracted from tumor and corresponding normal colonic tissues were subjected to STR analysis for MSI and 18q LOH status using a panel of 10 mononucleotide and dinucleotide microsatellite loci: *D2S123, D5S346, D17S250, BAT25, BAT26, BAT40, D18S55, D18S56, D18S67*, and *D18S487* [44, 80, 81]. The forward primer for each marker was labeled with fluorescence (either FAM or HEX) at the 5′ end (Supplementary Table S5, available online). Approximately 30–50 ng of gDNA was amplified in a 50-μL PCR reaction that contained 15 pmol of forward and reverse primers and 0.6 μL (5 U/μL) of KAPA Taq DNA Polymerase (Kapa Biosystems, USA). The thermocycling conditions were as follows: initial activation at 94°C for 3 minutes; 35 cycles of denaturation at 94°C for 25 seconds, annealing at 55°C for 25 seconds, and elongation at 72°C for 1.5 minutes; and a final extension at 72°C for 3 minutes. The PCR products were electrophoresed and analyzed using an ABI 3730XL DNA Analyzer (Applied Biosystems, USA) with GeneMarker V2.2.0 (SoftGenetics, LLC, USA).

The MSI status was graded as high (MSI-H; 3 or more unstable markers), low (MSI-L; 1 to 2 unstable markers), or stable (MSS; no unstable markers) [44]. The MSI-L and MSS populations were pooled. LOH at each locus in 18q (*D18S55*, *D18S56*, *D18S67*, and *D18S487*) was defined as *a* ≥ 40% reduction in 1 of 2 allele peaks in tumor DNA relative to normal DNA in two duplicate runs. A tumor was defined as 18q LOH positive when any informative marker showed LOH; and negative when at least two markers were informative and the absence of LOH [33].

### Statistical analysis

Kolmogorov-Smirnov *Z* tests were performed to test whether the LMRs were normally distributed according to various grouping factors. The student *t* test was used to compare the mean LMRs between the two independent populations when the data was normally distributed, otherwise the Mann-Whitney *U* test were utilized. Chi-square test was utilized to compare differences between two observed frequencies. The cut-off of the LMRs was calculated using the X-tile program (http://www.tissuearray.org/rimmlab/), which identified the cut-off value with minimum *p* values from log-rank x^2^ statistics for the categorical LMRs in terms of cancer specific survival [82–84]. This cut-off value was used to further subgroup the patients into low or high LMR levels. Cumulative survival curves were drawn using the Kaplan-Meier method, and the differences between the curves were analyzed by the log-rank test. Prognostic factors were determined using multivariate Cox regression analysis. Statistical analyses were performed using SPSS ver. 20.0 (IBM Corp., USA). A two-tailed *p* value less than 0.05 was considered statistically significant.

## SUPPLEMENTARY TABLES


