# Supplementary material for: LINE-1 hypomethylation in normal colon mucosa is associated with poor survival in Chinese patients with sporadic colon cancer
Source: Oncotarget. 2015 Jun 29;6(27):23820–36. doi: 10.18632/oncotarget.4450 (PMC4695154; doi:10.18632/oncotarget.4450)
Supplement: Supplementary file 1 [file oncotarget-06-23820-s001.pdf]

SUPPLEMENTARY TABLES

Supplementary Table S1: Kolmogorov-Smirnov *Z* tests for normality distribution of LINE-1 methylation rates

Variable, and grouping factor	*Z* value	*p* value	Normal distribution
Overall (one sample)	0.881	0.420	Yes
Two independent samples			
Sex, Male/Female	0.687	0.732	Yes
Age, ≤60/>60 yearsSize, ≤5/>5 cm	0.5910.521	0.8760.949	YesYes
Location, Left side/Right side	0.854	0.459	Yes
Mucinous or Signet ring carcinoma, No/Yes	0.720	0.677	Yes
Differentiation, G1-G2/G3-G4	0.520	0.950	Yes
Perineural invasion, No/Yes	0.899	0.393	Yes
Extranodal tumor deposits, No/Yes	1.062	0.210	Yes
Tumor stage, T2–T3/T4a–T4b	0.786	0.566	Yes
Nodal status, N0/N1–2	0.815	0.519	Yes
Distant metastases, M0/M1	0.795	0.552	Yes
AJCC Stage, I–II/ III–IV	0.856	0.456	Yes
Serum CEA level, Normal/Elevated	0.524	0.947	Yes
Serum CA199 level, Normal/Elevated	1.092	0.184	Yes
KRAS gene mutation, Wild type/Mutation	1.186	0.120	Yes
BRAF gene mutation, Wild type/Mutation	0.521	0.949	Yes
NRAS gene mutation, Wild type/Mutation	0.686	0.735	Yes
MS Status, MSI-L or MSS/MSI-H	0.707	0.699	Yes
18q LOH, No/Yes	0.778	0.580	Yes
hMLH1 promoter hypermethylation, No/Yes	0.663	0.771	Yes
hMSH2 promoter Hypermethylation, No/Yes	0.731	0.660	Yes
Lymphovascular invasion, No/Yes	1.412	0.037	No
PIK3CA gene mutation, No/Yes	1.350	0.496	No

*A two-tailed p value ≤ 0.05 was considered statistically significant. For normality distribution test, a population with a Z value > 1 and p value > 0.05 was considered as normally distributed.

[a]The left side of the colon consists of the splenic flexure, descending and sigmoid colon. The right side of the colon consists of the cecum, ascending colon, hepatic flexure, and transverse colon.

Abbreviations: AJCC, American Joint Committee on Cancer; CEA, carcinoembryonic antigen; CA199, Carbohydrate antigen 199; MSI, micro-satellite instable; MSS, micro-satellite stable; 18q LOH, loss of heterozygosity at chromosome 18q.

Supplementary Table S2: Chi-square analysis on patient subgroups according to *KRAS* gene mutation

KRAS gene mutation	Left-sided colon	Right-sided colon	X^2	*p*
Wild Type	40 (71.4%)	35 (49.3%)		
Mutation	16 (28.6%)	36 (50.7%)	6.342	0.012

Supplementary Table S3: Chi-square analysis on patient subgroups according to *hMLH1* promoter hypermethylation

hMLH1 promoter hypermethylation	MSI-L or MSS	MSI-H	X^2	*p*
No	87 (82.9%)	14 (63.6%)		
Yes	18 (17.1%)	8 (36.4%)	4.127	0.042

Supplementary Table S4: Stratification analysis for the influence of LMR level on patients' CCSS

Variables	Stratum	LMR level	EM (months)	SE	95% CI (Lower-upper)	X^2	*p* value
Age	>60 years	Low	47.964	6.482	35.260–60.668	7.953	0.005
		High	71.291	4.399	62.670–79.913		
Size	≤5cm	Low	58.293	5.485	47.543–69.044	4.293	0.038
		High	72.495	4.024	64.607–80.382		
Location [a]	Right-sided	Low	52.482	5.925	40.869–64.095	5.246	0.022
		High	69.816	4.586	60.828–78.804		
Distant metastases	M0	Low	74.277	3.773	66.882–81.673	5.140	0.023
		High	80.893	1.551	77.852–83.933		
Differentiation	G3–G4	Low	45.438	7.309	31.114–59.763	4.211	0.040
		High	66.701	6.452	54.055–79.346		
Perineural invasion	No	Low	60.359	4.737	51.074–69.644	6.232	0.013
		High	73.530	3.180	67.297–79.762		
Serum CEA level	Normal	Low	61.635	5.760	50.345–72.925	6.181	0.013
		High	76.256	3.204	69.975–82.536		
KRAS gene mutation	Mutation	Low	45.215	8.145	29.251–61.179	11.362	0.001
		High	74.456	4.087	66.446–82.466		
BRAF gene mutation	Wild type	Low	57.304	4.813	47.870–66.739	6.746	0.009
		High	71.341	3.265	64.942–77.741		

(*Continued*)

Variables	Stratum	LMR level	EM (months)	SE	95% CI (Lower-upper)	X^2	*p* value
PIK3CA gene mutation	Wild type	Low	57.414	4.679	48.243–66.585	5.006	0.025
		High	69.150	3.637	62.021–76.278		
18q LOH	Yes	Low	49.277	6.489	36.558–61.996	4.210	0.040
		High	65.682	4.626	56.615–74.748		
hMLH1 promoter	No	Low	54.402	5.242	44.129–64.676	4.939	0.026
hypermethylation		High	68.928	3.850	61.382–76.473		

*Log Rank (Mantel-Cox) test was used to test the significance of the different survival between the groups according to different variables. A two-tailed *p* value ≤0.05 was considered statistically significant.
[a]The left side of the colon consists of the splenic flexure, descending and sigmoid colon. The right side of the colon consists of the cecum, ascending colon, hepatic flexure, and transverse colon.
Abbreviations: CCSS, colon cancer specific survival; EM, estimated mean; SE, standard error; CI: confidence interval; LMR, LINE-1 methylation rate; 18q LOH, loss of heterozygosity at chromosome 18q; MSI, micro-satellite instable; MSS, micro-satellite stable; CEA, carcinoembryonic antigen; CA199, Carbohydrate antigen 199; AJCC, American Joint Committee on Cancer (AJCC).

Supplementary Table S5: Student's *t* test for the LMRs in various variables

Variables	Sub-groups	Mean	SE	*t*	*p*
Sex	Male	64.7722	1.34628	0.196	0.845
	Female	64.3416	1.61891		
Age	≤60 years	63.8597	1.75391	−0.716	0.476
	>60 years	65.3511	1.15971		
Size	≤5 cm	64.4210	1.12707	−0.337	0.736
	>5 cm	65.0823	1.72282		
Location[a]	Left-sided	64.2400	1.58302	−0.326	0.745
	Right-sided	64.9251	1.38740		
Mucinous or Signet ring carcinoma	No	64.4210	1.12707	−0.391	0.696
	Yes	65.4472	2.64325		
Differentiation	G1–G2	64.6427	1.37045	0.024	0.981
	G3–G4	64.5916	1.59979		
Perineural invasion	No	64.2076	1.18164	0.983	0.328
	Yes	67.1383	1.54461		
Extranodal tumor deposits	No	64.8435	1.10761	−0.504	0.615
	Yes	63.3695	3.00469		
Tumor stage (T)	T2–T3	66.4479	1.29345	1.493	0.138
	T4a–T4b	63.3159	1.51421		

(*Continued*)

Variables	Sub-groups	Mean	SE	*t*	*p*
Nodal status (N)	N0	63.5153	1.40564	−0.992	0.323
	N1–2	65.5841	1.51109		
Distant metastases (M)	M0	64.6064	1.25442	.027	.979
	M1	64.6703	1.83180		
AJCC Stage	I–II	65.3466	1.64836	0.668	0.506
	III–IV	63.9542	1.30390		
Serum CEA level	Normal	64.8810	1.36081	−0.312	0.756
	Elevated	64.2122	1.61891		
Serum CA199 level	Normal	62.7563	1.73554	−1.242	0.216
	Elevated	65.5129	1.28917		
KRAS gene mutation	Wild type	62.9409	1.39699	1.965	0.052
	Mutation	67.0490	1.49789		
BRAF gene mutation	Wild type	66.7860	3.51702	0.420	0.675
	Mutation	64.5343	1.07401		
NRAS gene mutation	Wild type	64.6612	1.05439	−0.235	0.814
	Mutation	63.0433	7.48089		
MSI Status	MSI-L or MSS	65.0744	0.98691	−0.948	0.345
	MSI-H	62.4686	3.76247		
18q LOH	No	63.4533	1.96858	0.921	0.359
	Yes	65.4079	1.13176		
hMLH1 promoter hypermethylation	No	64.8089	1.21354	−0.351	0.726
	Yes	63.9008	1.92494		
hMSH2 promoter Hypermethylation	No	64.7881	1.15190	−0.377	0.707
	Yes	63.6847	2.38576		

*A two-tailed p value ≤ 0.05 was considered statistically significant.

[a]The left side of the colon consists of the splenic flexure, descending and sigmoid colon. The right side of the colon consists of the cecum, ascending colon, hepatic flexure, and transverse colon.

Abbreviations: AJCC, American Joint Committee on Cancer; CEA, carcinoembryonic antigen; CA199, Carbohydrate antigen 199; MSI, micro-satellite instable; MSS, micro-satellite stable; 18q LOH, loss of heterozygosity at chromosome 18q.

Supplementary Table S6: Primers sequences used in this study

Gene	Primer sequence	Production size (bp)
**Pyrosequencing PCR for LINE-1 methylation status[1]**		
LINE-1	Forward: 5′-TGGTTAAGGGTTTGGGGATATT-3′<br>Reverse: 5’-Biotin-AACACAATTCCCAACCCAC-3′<br>Sequencing: 5′-AACACAATTCCCAACCCAC-3′	88
**MS-qPCR for *hMHL1* and *hMSH2* gene promoter methylation level[2, 3]**		
hMHL1	Forward: 5′-ACGTAGACGTTTTATTAGGGTCGC-3’<br>Reverse: 5′-CCTCATCGTAACTACCCGCG-3′	100
hMSH2	Forward: 5′-TCGTGGTCGGACGTCG TTC-3′<br>Reverse: 5′-CAACGTCTCCTTCGACTACACCG-3′	121
ALU-C4	Forward: 5′-GTTAGGTATAGTGGTTTATATTTGTAATTTTAGTA-3′<br>Reverse: 5′-ATTAACTAAACTAATCTTAAACTCCTAACCTCA-3′	98
**STR analysis for MSI and 18q LOH status[4, 5]**		
BAT25	Forward: 5′-FAM-TCGCCTCCAAGAATGTAAGT- 3′<br>Reverse: 5′-TCTGCATTTTAACTATGGCTC-3′	110–130
BAT26	Forward: 5′-HEX-TGACTACTTTTGACTTCAGCC-3′<br>Reverse: 5′-AACCATTCAACATTTTTAACCC-3′	100–120
D2S123	Forward: 5′-FAM-AAACAGGATGCCTGCCTTTA-3′<br>Reverse: 5′-GGACTTTCCACCTATGGGAC-3′	197–227
D5S346	Forward: 5′-HEX-AGCAGATAAGACAGTATTACTAGTT-3′<br>Reverse: 5′-ACTCACTCTAGTGATAAATCGGG-3′	100–130
D17S250	Forward: 5′-FAM-GGAAGAATCAAATAGACAAT-3′<br>Reverse: 5′- GCTGGCCATATATATATTTAAACC–3′	150–160
BAT40	Forward: 5′-HEX-AGCCAAGATTAACTTCCTACACCACAAC-3′<br>Reverse: 5′-GTAGAGCAAGACCACCTTGTCTC-3′	120–140
D18S55	Forward: 5′-FAM-GTGTCTTCAATATTGATTCTCTATTCTAGCCT-3′<br>Reverse: 5′-AGCTTCTGAGTAATCTTATGCTGTG-3′	100–120
D18S56	Forward: 5′-HEX-GTGTCTTCCTGAAGGACCTGCCTGAGATA-3′<br>Reverse: 5′-CTATACTTTTTATTGTTAGGGTGTG-3′	95–115
D18S67	Forward: 5′-FAM-CTTGGGTTCCATCTTCAGGAAA-3′<br>Reverse: 5′-GTGTCTTATGAGATAGGCCCAAAGCATC-3′	95–120
D18S487	Forward: 5′-HEX-GTGTCTTGCCAAATTAAAAGAATGTATATTATT GC-3′<br>Reverse: 5′-GATTTTCCTCGTGCGTGCTT-3′	85–105

Supplementary Table S7: Point mutations analysis and the primer sets for targeted genes

Gene	Possible DNA point mutation	Associated Codon and AA alteration	Associated Exon	Primer sets (5′ to 3′)
BRAF	c.1798_1799GT>AA	p.Val600Lys	15	Nest PCRs<br>F1: GACTCTAAGAGGAAAGATG<br>R1: ACTGATTTTTGTGAATACTG
	c.1798G>A	p.Val600Met		
	c.1798G>T	p.Val600Leu		
	c.1799T>A	p.Val600Glu		F2: CTTCATAATGCTTGCTCTGATAGG
	c.1799T>C	p.Val600Ala		R2: AGCCTCAATTCTTACCATCCAC
	c.1799T>G	p.Val600Gly		
KRAS	c.34G>A	p.Gly12Ser	2	1F: TCTTAAGCGTCGATGGAGG<br>1R: AAGAATGGTCCTGCACCAG
	c.34G>C	p.Gly12Arg		
	c.34G>T	p.Gly12Cys		
	c.35G>A	p.Gly12Asp		
	c.35_36delinsCA	p.Gly12Ala		
	c.35G>C	p.Gly12Ala		
	c.35G>T	p.Gly12Val		
	c.37G>A	p.Gly13Ser		
	c.37G>C	p.Gly13Arg		
	c.37G>T	p.Gly13Cys		
	c.37G>C	p.Gly13Arg		
	c.38G>A	p.Gly13Asp		
	c.38G>C	p.Gly13Ala		
	c.38G>T	p.Gly13Val		
	c.180_181delinsAA	p.Gln61Lys	3	2F: CCGTCATCTTTGGAGCAGG<br>2R: TACCGATGCAGTCTGGAGC
	c.181C>A	p.Gln61Lys		
	c.181C>G	p.Gln61Glu		
	c.181C>T	p.Gln61X		
	c.182A>C	p.Gln61Pro		
	c.182A>G	p.Gln61Arg		
	c.182A>T	p.Gln61Leu		
	c.183A>C	p.Gln61His		
	c.183A>T	p.Gln61His		
	c.436G>A	p.Ala146Thr	4	3F: CACAAAACAGGCTCAGGACTT<br>3R: GCCAAAAGCAGTACCATGGA
	c.436G>C	p.Ala146Pro		
	c.437C>T	p.Ala146Val		
NRAS	c.34G>A	p.Gly12Ser	2	1F: TGTAGATGTGGCTCGCCAA<br>1R: CGCTACTATGGCCTGTGTT

(*Continued*)

Gene	Possible DNA point mutation	Associated Codon and AA alteration	Associated Exon	Primer sets (5′ to 3′)
	c.34G>C	p.Gly12Arg		
	c.34G>T	p.Gly12Cys		
	c.35G>A	p.Gly12Asp		
	c.35G>C	p.Gly12Ala		
	c.35G>T	p.Gly12Val		
	c.37G>A	p.Gly13Ser		
	c.37G>C	p.Gly13Arg		
	c.37G>T	p.Gly13Cys		
	c.38G>A	p.Gly13Asp		
	c.38G>C	p.Gly13Ala		
	c.38G>T	p.Gly13Val		
	c.181C>A	p.Gln61Lys	3	2F: GCAATTTGAGGGACAAACCA<br>2R: CTCCTAGTACCTGTAGAGGTT
	c.181C>G	p.Gln61Glu		
	c.181C>T	p.Glu61X		
	c.182A>C	p.Gln61Pro		
	c.182A>G	p.Gln61Arg		
	c.182A>T	p.Gln61Leu		
	c.183A>C	p.Gln61His		
	c.183A>T	p.Gln61His		
PIK3CA	c.1624G>A	p.Glu542Lys	9	1F: TCTGTGAATCCAGAGGGGA<br>2R: AACATGCTGAGATCAGCCA
	c.1624G>C	p.Glu542Gln		
	c.1633G>A	p.Glu545Lys		
	c.1633G>C	p.Glu545Gln		
	c.1636C>A	p.Gln546Lys		
	c.1636C>G	p.Gln546Glu		
	c.1637A>C	p.Gln546Pro		
	c.1637A>G	p.Gln546Arg		
	c.1637A>T	p.Gln546Leu		
	c.3139C>T	p.His1047Tyr	20	2F: CTTGGCTCTGGAATGCCAG<br>2R: ATGCAATCGGTCTTTGCCT
	c.3140A>G	p.His1047Arg		
	c.3140A>T	p.His1047Leu		
	c.3145G>A	p.Gly1049Ser		
	c.3145G>C	p.Gly1049Arg		

Abbreviations: AA, amino acid; BRAF, v-raf murine sarcoma viral oncogene homolog B; KRAS, Kirsten rat sarcoma viral oncogene homolog; NRAS, neuroblastoma RAS viral oncogene homolog; PIK3CA, phosphatidylinositol-4, 5-bisphosphate 3-kinase, catalytic subunit a.

REFERENCES

1. Hur K, Cejas P, Feliu J, et al. Hypomethylation of long interspersed nuclear element-1 (LINE-1) leads to activation of proto-oncogenes in human colorectal cancer metastasis. Gut. 2014; 63:635–646.
2. Farris AB 3rd, Demicco EG, Le LP, et al. Clinicopathologic and molecular profiles of microsatellite unstable Barrett Esophagus-associated adenocarcinoma. Am J Surg Pathol. 2011; 35:647–655.
3. Weisenberger DJ, Campan M, Long TI, et al. Analysis of repetitive element DNA methylation by MethyLight. Nucleic Acids Res. 2005; 33:6823–6836.
4. Wooster R, Cleton-Jansen AM, Collins N, et al. Instability of short tandem repeats (microsatellites) in human cancers. Nat Genet. 1994; 6:152–156.
5. Ogino S, Brahmandam M, Cantor M, et al. Distinct molecular features of colorectal carcinoma with signet ring cell component and colorectal carcinoma with mucinous component. Mod Pathol. 2006; 19:59–68.
